# Hypergravity induced disruption of cerebellar motor coordination

**DOI:** 10.1038/s41598-020-61453-w

**Published:** 2020-03-10

**Authors:** Wonjun Noh, Minseok Lee, Hyun Ji Kim, Kyu-Sung Kim, Sunggu Yang

**Affiliations:** 1 Department of Nano-Bioengineering, Incheon National University, Incheon, Korea; 2 Department of Otorhinolaryngology-Head & Neck surgery, Inha University, College of medicine, Incheon, Korea

**Keywords:** Cellular neuroscience, Ion channels in the nervous system, Cerebellum

## Abstract

The cerebellum coordinates voluntary movements for balanced motor activity in a normal gravity condition. It remains unknown how hypergravity is associated with cerebellum-dependent motor behaviors and Purkinje cell’s activities. In order to investigate the relationship between gravity and cerebellar physiology, we measured AMPA-mediated fast currents and mGluR1-mediated slow currents of cerebellar Purkinje cells along with cerebellum-dependent behaviors such as the footprint and irregular ladder under a hypergravity condition. We found abnormal animal behaviors in the footprint and irregular ladder tests under hypergravity. They are correlated with decreased AMPA and mGluR1-mediated synaptic currents of Purkinje cells. These results indicate that gravity regulates the activity of Purkinje cells, thereby modulating cerebellum-dependent motor outputs.

## Introduction

Gravity shift is accompanied by various biological complications: (1) Reduction of cardiac parasympathetic activity and spontaneous arterial-cardiac baroreflex function^1^; (2) Motion sickness symptoms such as pallor, increased body warmth, cold sweating, malaise, loss of appetite, nausea, fatigue, vomiting, and anorexia^2–4^; (3) Reduced red cell’s mass/plasma volume and orthostatic intolerance^5,6^. Animals exposed to hypergravity have the deleterious effect of a muscle function and show a higher anxiety level^7^. In the genetic level, gene expression pattern is differently regulated depending on brain regions under different gravity conditions. Hypergravity increases the expression of several hippocampus-related genes such as proSAAS, Neuroblastoma ras oncogene, Thymosin beta-10, Inhibin beta E, Ngfi-A binding protein 2 and Syndet while microgravity decreases genes as phosphatidylethanolamine binding protein and Pgam 1 protein^8,9^. Also, brain-derived neurotrophic factor (BDNF) is decreased in the ventral hippocampus and hypothalamus whereas increased in the cerebellum. Serotonin receptors (5-HT1BRs) are decreased in the cerebellum whereas increased in the ventral hippocampus and caudate putamen^10^. Hypergravity severely affects cerebellar functions, leading to dysfunction of motor coordination. It is also accompanied by the decreased number of Purkinje cells, cerebellar mass and cerebellar proteins such as GFAP and synaptophysin^11–13^. For now, a cellular mechanism underlying those changes remains uncertain.

mGluR1 expressed in Purkinje cells plays a critical role in motor coordination^14–18^. mGluR1-deficient mice have ataxia that can be rescued by selective expression of mGluR1 in Purkinje neurons^19^. mGluR1 is activated by repetitive stimulation at parallel fiber synapses, mediating a signaling cascade of mGluR1-mediated G-proteins such as Gaq, Ga11, and phospholipase Cb (PLC-b)^20^. Also, mGluR1 activates nonselective cation channels such as TRP1^16^ or TPRC3^18^ which induces slow excitatory postsynaptic potentials (EPSPs)^16,18,21^. Furthermore, mGluR1 activation to repetitive, strong stimulation on parallel fibers produces Ca^2+^ influx from intracellular stores along with the TRPC3-mediated slow currents^20,22^. Meanwhile, weak stimulation on parallel fibers only induces AMPA-mediated fast currents. Recently, there are growing interest of those currents being involved in animal behaviors. In particular, the malfunction of mGluRs has been a root cause of many brain diseases ^23–26^.

How the hypergravity influence on AMPAR-/mGluR-mediated current and motor coordination has not been demonstrated. Here, we investigated Purkinje cell’s intrinsic properties and mGluR1- and AMPAR-mediated currents-associated with motor coordination under the condition of hypergravity. Our research goal is to study cellular mechanisms of hypergravity-induced disruption of cerebellar motor coordination, providing scientific perspective for gravity shift (e.g. flight to the space and back to earth).

## Method

### Hypergravity exposure

**Figure 1:**
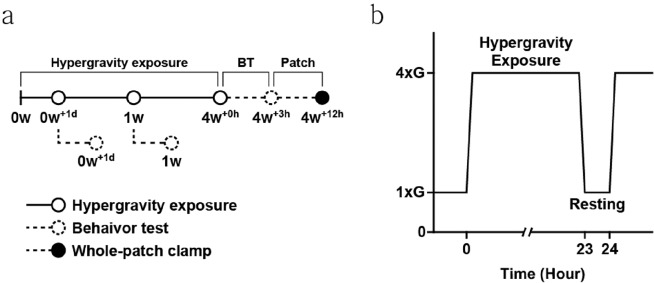
The schematic of hypergravity exposure. (**a**) Experimental schematic for the hypergravity induction. SD rats are exposed under hypergravity for 1 day, 1 week and 4 weeks. Right after that, behavior and electrophysiology experiments are performed. (**b**) 24 h timetable for hypergravity exposure. 23 h’ hypergravity exposure followed by an hour rest period.

7 weeks old male SD rats started to be conditioned by in a house-designed, gravitational force (G-force) simulator with two horizontal rotatory arms (50 cm long each). The simulator is a long dumbbell-like rod in which an animal cage can be held in each end. When the arms are rotated, centrifugal force is exported on the animal cage, which is suspended from the arms end. When the arms rotate at a speed of 65 rpm, the rats in the cage are exposed to 4 G hypergravity. A high-resolution video camera, which was placed inside the cage, was used to evaluate whether the mice could move freely, and access food and water. The rats were exposed to HG for 23 hours and took an hour rest time under normal gravity (1 G). The conditioning process was repeated for 4 weeks (Fig. 1a). Hypergravity exposure process is continued twenty-three hours a day with one hour resting for 4 weeks (Fig. 1b). The behavior tests followed by electrophysiology were conducted right after hypergravity exposure.

### Slice preparation

All animal handling procedures were approved by the Institutional Animal Care and Use Committee of Inha University and Incheon National University. Animals were treated in accordance with the National Institutes of Health Guide for the care and use of laboratory animals, and the Animal Welfare Act (7 U.S.C. et seq.). Sprague-Dawley rat (postnatal age: 11 weeks) for brain slicing were deeply anesthetized with halothane. The Brains were quickly removed and placed into chilled (4 °C), oxygenated (5% CO_2_ and 95% O_2_) slicing medium containing (in mM): 110 Choline Chloride, 2.5 KCl, 1.2 NaH_2_PO_4_, 25 NaHCO_3_, 20 glucose, 7 MgCl_2_, 0.5 CaCl_2_. Sagittal slices (300 μm) were cut using vibratome. Rat Brain Slices were then transferred to a holding chamber containing oxygenated physiological saline made up of (in mM): 124 NaCl, 2.5 KCl, 1 NaH_2_PO_4_, 26.2 NaHCO_3_, 20 glucose, 1.3 MgCl_2_, 2.5 CaCl_2_. After at least 1 h recovery, individual slices were transferred to a recording chamber. Oxygenated physiological saline was continuously superfused at a rate of 1.5 ml/min at 32–33 °C temperature.

### Electrophysiological recordings

Whole-cell patch recordings were obtained using an Axon instruments Axoclamp 700B Amplifier (Molecular Devices), and recording pipettes had tip resistances of 4–6 MΩ when filled with a solution containing (in mM): 135 Cesium methanesulfonate, 10 CsCl, 0.2 EGTA, 10 HEPES, 4 Na_2_-ATP, 0.4 Na-GTP. The pH and osmolality of intracellular solution were adjusted to 7.3 and 290 mOsm, respectively. Recordings were done in current-clamp configuration and cells were held at −65 mV: the resting membrane potential was in the range of −65 to −70 mV after whole-cell configuration. During recordings, an access resistance was continually monitored. Recordings were excluded if an input resistance changed by>15%. pClamp Version 10.2 software (Molecular Devices) was used for data acquisition. Whole-cell patch recordings were made on Purkinje neurons. Give 1 pulse (10 ms duration) and 5 pulses (50 ms duration, 100 Hz, 1 ms pulse width) stimulation. During the recordings, the slices were continuously remained at 32–33 °C temperature with ACSF that contained 10μM SR (TOCRIS), 2μM NBQX (TOCRIS).

### Behavior test

**Figure 2:**
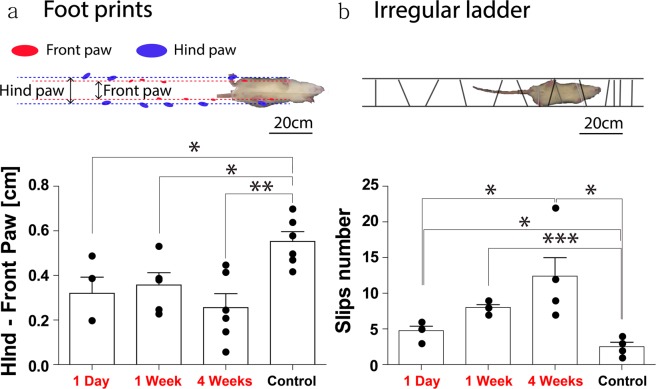
Disruption of motor coordination under HG. (**a**) Illustration of a footprints test. The image shows a rat walking on a glass plate. Dotted lines depict footprints of front (red) and hind (blue) paws. Distance differences between front and hind paws are measured in both control and HG groups with different exposure times (1 Day, 1 week and 4 weeks). (**b**) The image shows a rat walking on a ladder which is inclined (30~40°) and has irregular rods. Slipping numbers are counted when a rat walks across in both control and HG rats with different exposure times (1 Day, 1 week and 4 weeks) (all data are normalized as student’s *t*-test. ***P < 0.001. **P < 0.005. *P < 0.05.).

In order to measure animals’ capability of motor coordination under gravity shift, we forced control and HG-exposed rats to perform a footprint and irregular ladder test (see Fig. 2 for illustration of behavior tests). As for the footprint test, we made a long, foot-printable glass plate (with 10 cm width and 200 cm length) where the rats can walk along. Also, they were tested in 1 day, 1 week and 4 weeks so as to measure effects of HG over time. While rats were walking on the glass plate from the beginning to the end, we measured the lateral distance between front and hind paws in both control and HG groups with the different exposure times. The result was analyzed according to the distance pattern of footprint on the walking pad. As for the irregular ladder test, control and HG groups were allowed to walk on a zigzag patterned ladder which challenges rats to balance and walk straightly. While rats were climbing on the irregular ladder from the bottom to top, slipping numbers were counted in both control and HG rats with different exposure times.

### Data analysis

**Figure 3:**
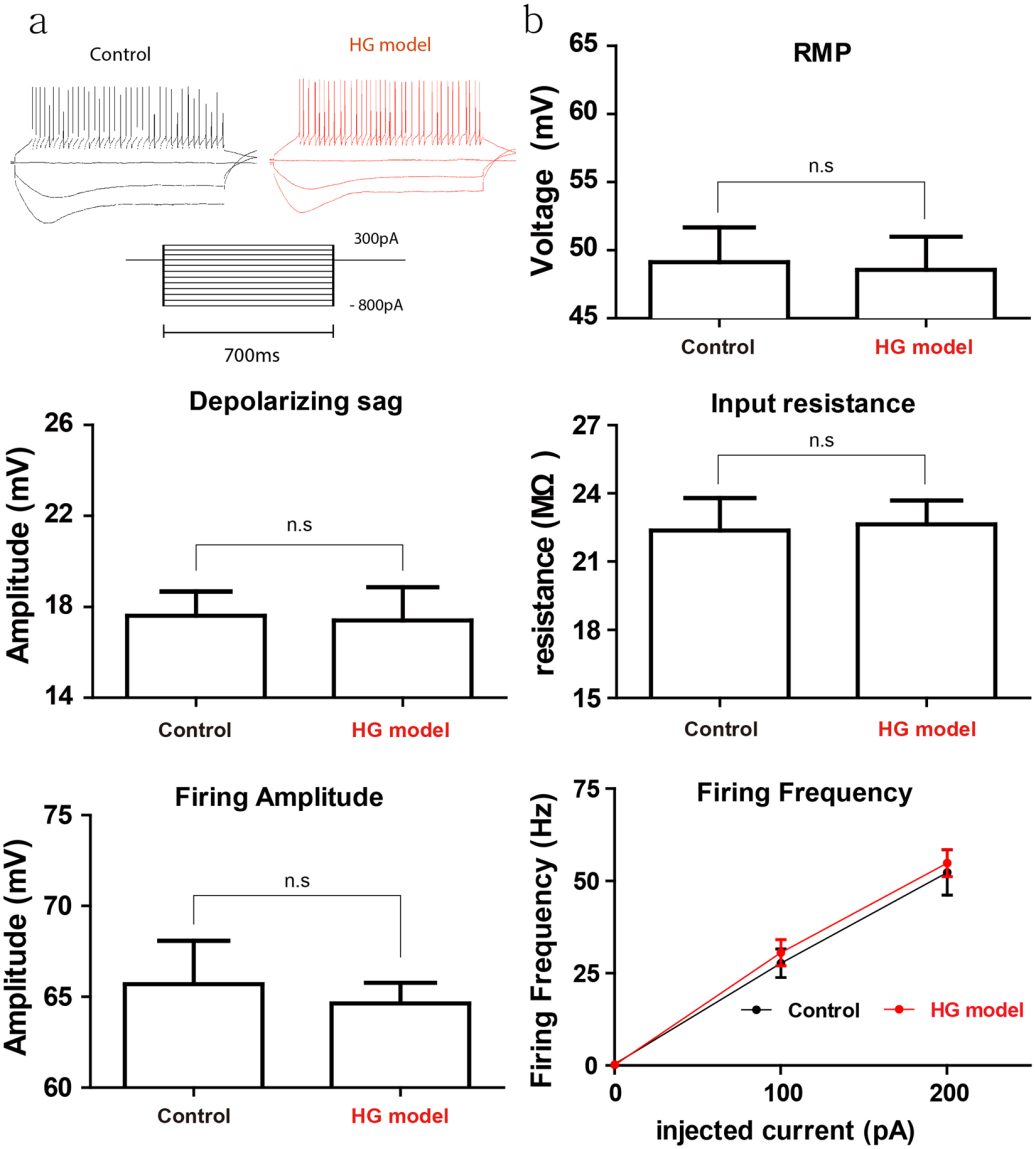
Unaltered Purkinje cell’s intrinsic properties under hypergravity. (**a**) Responses to long depolarizing and hyperpolarizing currents (700 ms) from −800 pA to 300 pA have a firing pattern and hyperpolarization-activated current (Ih)-mediated depolarization sag, respectively. (**b**) Cells’ intrinsic properties in resting membrane potential (RMP), input resistance, depolarizing sag, firing amplitude and I/O function (e.g., firing frequency over injected current). There are no differences between the control and hypergravity group. n.s, not significant.

**Figure 4:**
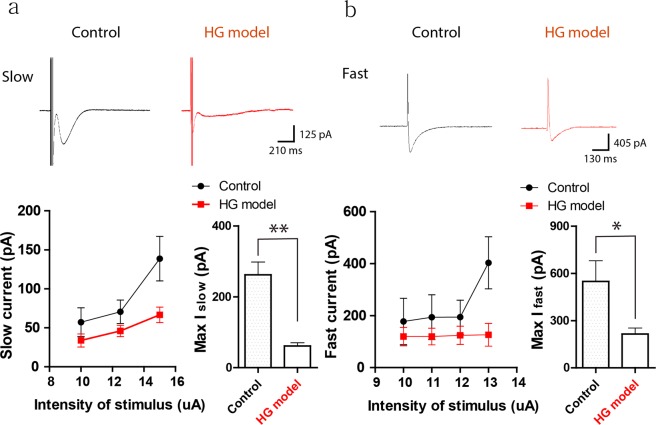
Decreased I_*slow*_ and I_*fast*_ in the hypergravity condition. (**a**) Burst stimulation of Parallel fibers produces mGluR1-mediated slow currents (under voltage-clamp mode; resting membrane potential, −65 mv). According to two-way ANOVA, slow currents (over various intensities) is reduced under hypergravity and Student’s t-test showed that Max I_*slow*_ currents are reduced under hypergravity. (**b**) Single stimulation of Parallel fibers produces AMPAR-mediated fast currents. According to two-way ANOVA, fast currents (over various intensities) is reduced under hypergravity and Student’s t-test showed that Max I_*fast*_ currents are reduced under hypergravity. **p < 0.005. *p < 0.05.

All electrophysiological data are treated and expressed numerically using Axon^TM^ pCLAMP^TM^11 Electrophysiology Data Acquisition & Analysis Software (Molecular Devices, San Jose, CA). The student’ t-test in Fig. 3 was used for statistical significance while two-way analysis of variance (ANOVA) in Fig. 4 was used to assess statistical significance for the amplitude of slow current and fast current as a function of the stimulus intensity. For the differences between HG and control group, P < 0.05 was interpreted statistically significant (*P < 0.05, **P < 0.005, ***P < 0.001). All Graphs were prepared in GraphPad® Prism 7 (GraphPad Software Inc., La Jolla, CA) and final arrangement and labeling were carried out using Adobe illustrator CC 2019 (Adobe Inc., San Jose, CA). All data are illustrated as mean ± Standard Error of the Mean (SEM). n indicates the number of animals in behavior tests, and slices in EPG.

## Result

In order to test whether hypergravity affects motor coordination, we examined a footprint test and irregular ladder which are known to be indicators of how animals coordinate motor skills. When rats walked on a glass plate, we measured the average distance of their footprints between each side of hind and front paw both control and hypergravity groups defined as 1 day, 1 week, and 4 week (Fig. 2a). Footprint distance of 4 week HG group had smaller distance than that of the control group (Fig. 2a, Control: 0.553 ± 0.043, n = 6; 4 weeks: 0.256 ± 0.062, n = 6, **P = 0.0029). Similarly, we found that 1 day and 1 week HG groups had significant reduction in footprints test compared to control group (Fig. 2a, Control: 0.553 ± 0.043, n = 6; 1 day: 032 ± 0.072, n = 4, *P = 0.0187; 1 weeks: 0.553 ± 0.044, n = 5, *P = 0.0202). Meanwhile there is no significance between 1 day, 1 week and 4 weeks HG groups (Fig. 2a, 1 day: 0.32 ± 0.072, n = 4; 1 weeks: 0.356 ± 0.054 n = 5; 4 weeks: 0.256 ± 0.062, n = 6).

An Irregular ladder test was conducted by counting the number of slip when rats were walking on a ladder (Fig. 2b). The slip number of the 4 weeks HG group was larger than that of the control group (Fig. 2b, Control: 2.5 ± 0.64, n = 4; 4 weeks: 12.4 ± 2.58, n = 6, *P = 0.0127). Similarly, we found that slip number of the 1 day and 1 week HG group was larger than that of the control group (Fig. 2b, 1 day: 4.75 ± 0.63, n = 4; 1 week: 8 ± 0.41, n = 4; Control: 2.5 ± 0.64, n = 4, *P = 0.047). The slip number of the 1 days HG groups was smaller than that of the 4 weeks HG groups (Fig. 2b, 1 day: 4.75 ± 0.63, n = 4; 4 weeks: 12.4 ± 2.58, n = 6, *P = 0.037). But, there is no significance between 1 week and 4 weeks HG groups (Fig. 2b, 1 week: 8 ± 0.41, n = 4; 4 weeks: 12.4 ± 2.58, n = 5). The altered front-hind footprint distance and improper stepping indicate hypergravity-induced disruption of motor coordination.

Next, we wondered whether the hypergravity-induced impairment of motor coordination is associated with altered intrinsic membrane properties of Purkinje cells. For recording, we selected Purkinje cells in the lobule 9 around the posterior vermis of the cerebellum related to gravity and balance of gravity and balance^27–29^. We measured cells’ resting membrane potential (RMP), Input resistance (MΩ), depolarizing sec (mv), firing frequency (Hz) which are indicators of static membrane property of quiescent cells, signal holding capability, hyperpolarized-induced rebound action of signals, intrinsic firing behavior, respectively. We found there were no differences between the control and hypergravity group in resting membrane potential (Fig. 3b, Control: 49.12 ± 3.53 mv, n = 6; HG: 48.56 ± 2.97 mv, n = 6), input resistance (Control: 22.37 ± 1.43 MΩ, n = 5; HG: 22.64 ± 1.05 MΩ, n = 5), hyperpolarization-activated current (Ih)-mediated sag (Control: 17.61 ± 1.06 mv, n = 5, HG: 17.3 ± 1.33 mv, n = 5), firing amplitude (Control: 66.09 ± 2.66 mv, n = 5; HG: 64.63 ± 1.13 mv, n = 6) and, firing frequency/injected current slope (Control, 100 pA: 27.71 ± 3.85, n = 5; 200 pA: 52.29 ± 6.15, n = 5; HG, 100 pA: 30.57 ± 3.55, n = 5; 200 pA: 54.86 ± 3.69, n = 5). Our data showed that hypergravity does not affect Purkinje cell’s intrinsic property *per se*.

Then, we questioned whether cells’ synaptic properties are related to hypergravity. Purkinje cells have synaptic inputs from parallel fibers which are largely shaped by both mGluR1-dependent slow currents and AMPAR-dependent fast currents. We examined mGluR1-mediated slow (*I*_*slow*_) and AMPAR-mediated fast (*I*_*fast*_) currents by stimulating parallel fibers. There was significant reduction of *I*_*slow*_ amplitude over various intensities in hypergravity rats (Fig. 4a: Control 10 μA: 57.125 ± 18.46, 12.5 μA: 70.51 ± 15.11, 15 μA: 138.68 ± 28.52, n = 7; HG 10 μA: 33.74 ± 8.34, 12.5 μA: 45.9 ± 7.23, 115 μA: 66.58 ± 9.85, n = 6. F_(1,29)_ = 10.347, **P = 0.0032). I_fast_ amplitude in hypergravity rats was also reduced (Fig. 4b: Control 10 μA: 117.55 ± 89.15, 11 μA: 194 ± 86, 12 μA: 194.08 ± 65.34, 13 μA: 403.07 ± 100.06, n = 5; HG 10 μA: 119.48 ± 35.46, 11 μA: 119.583 ± 32.03, 12 μA: 124.32 ± 35.18, 13 μA: 126.2 ± 43.83, n = 6. F_(1,30)_ = 7.884, **P = 0.009). Next, we measured MAX *I*_*slow*_ in the control and hypergravity groups. MAX *I*_*slow*_ in the hypergravity group was smaller than that in the control group (Fig. 4a, Control: 261.93 ± 36.25, n = 7; HG: 61.18 ± 9.03, n = 9. **P = 0.0012). Also, MAX *I*_*fast*_ in the hypergravity group was smaller than that in control rats (Fig. 4b, Control: 548.63 ± 131.73, n = 6; HG: 215.01 ± 37.68, n = 9. *P = 0.0126). This result suggests that hypergravity modulates mGluR1- and AMPAR- dependent currents of Purkinje cells, which likely affects motor coordination.

## Discussion

In our studies, hypergravity impairs motor coordination which is accompanied by reduced mGluR1 and AMPA medicated currents of Purkinje cells. Meanwhile, the intrinsic properties of Purkinje’s cells are unaffected. The alteration of gravity negatively might influence neurotransmitter release in synapses of parallel fibers to Purkinje cells. Purkinje cells’ activity largely is influenced by two factors: 1) Intrinsic properties and 2) synaptic properties. There is no alteration in action potential generation under hypergravity as the cell’s intrinsic properties including firing frequency and amplitude are not altered. In contrast, HG can reduce mGluR- and AMPAR-mediated currents of Purkinje cells, which is caused by sluggish release of glutamate in the presynaptic terminals of parallel fibers and/or altered kinetics and/or sensitivity of both mGluRs and AMPARs in the postsynaptic Purkinje cells. A previous study showed HG alteres neurotransmitter reuptake by presynaptic transporters^30^. Nonetheless, we suggest that HG primarily affects postsynaptic mechanisms such as kinetics and/or expression of mGluRs and AMPARs. A further study is required to reveal whether presynaptic mechanisms are involved in the altered condition of gravity.

The central function of cerebellum is to build a dynamic prediction (e.g., an internal model) of the self-motion to ensure postural and perceptual stability^31,32^. The internal model allows cerebellum to maintain body movement against gravity. Gravity is a constant force that we experience through our entire lives. Thus, it is a neural mechanism shaped by balanced consequence of adaptation and stabilization through a learning process^33–37^. It is generally assumed according to a previous notion that the brain’s internal model is required to enforcedly adapt to the zero-G environment in outer space such that active motion generates an erroneous mismatch after re-entry^38^. For example, astronauts could experience disturbances in their internal balance when launching to and returning from earth under HG and staying in the outer space under MG. The exposure of different gravity could drive them to unlearn and later relearn the earth environment like the jet lag. As the learning process is closely associated with neural plasticity, there can be a potential contribution of gravity of long term potentiation/depression, a suggested cellular mechanism underlying learning, which is involved in Ca^2+^-permitting channels/receptors including NMDARs and mGluRs. Interestingly, we found hypergravity-induced disruption of AMPA and mGluR1 currents can be a molecular mechanism of such a mismatch underlying unlearning and relearning processes. In our experimental paradigm, there is a potential to affect synaptic transmission due to the abnormal animal behavior in rotation which can restrict normal access of food, water and space. Thus, the altered cerebellar synaptic activities under HG can be comorbid with abnormal animal welfare. Although the similar condition to astronauts can be considered, a further study to investigate the compounding effects seems to be required.

In conclusion, the Purkinje cells of cerebellum play an important role in regulating balanced movements as they provide inhibitory tones to motor network. Purkinje cells might be required to increase the inhibitory interference for stabilizing circuit excitability at the beginning of hypergravity. Purkinje cell’s synaptic activity can be decreased during long-term exposure of hypergravity as an adaptation mechanism. This negative correlation between cerebellum activity and gravity might provide important strategies of how to alleviate adverse symptoms when a gravity shift occurs in certain environments (e.g. trip to space and back to earth).

## References

[1] Iwasaki K (2005). Hypergravity exercise against bed rest induced changes in cardiac autonomic control. Eur. J. Appl. Physiol..

[2] Heer M, Paloski WH (2006). Space motion sickness: incidence, etiology, and countermeasures. Auton. Neurosci..

[3] Mucci V (2018). Mal de Debarquement Syndrome: a survey on subtypes, misdiagnoses, onset and associated psychological features. J. Neurol..

[4] Roberts DR (2017). Effects of Spaceflight on Astronaut Brain Structure as Indicated on MRI. N. Engl. J. Med..

[5] Buckey JC (1996). Orthostatic intolerance after spaceflight. J. Appl. Physiol..

[6] Alfrey CP, Udden MM, Leach-Huntoon C, Driscoll T, Pickett MH (1996). Control of red blood cell mass in spaceflight. J. Appl. Physiol..

[7] Bojados M, Jamon M (2014). The long-term consequences of the exposure to increasing gravity levels on the muscular, vestibular and cognitive functions in adult mice. Behav. Brain Res..

[8] Del Signore A (2004). Hippocampal gene expression is modulated by hypergravity. Eur. J. Neurosci..

[9] Sarkar P (2006). Proteomic analysis of mice hippocampus in simulated microgravity environment. J. Proteome Res..

[10] Ishikawa C (2017). Effects of gravity changes on gene expression of BDNF and serotonin receptors in the mouse brain. PLoS one.

[11] Nguon K, Li GH, Sajdel-Sulkowska EM (2004). CNS development under altered gravity: cerebellar glial and neuronal protein expression in rat neonates exposed to hypergravity. Adv. Space Res..

[12] Nguon K, Ladd B, Sajdel-Sulkowska EM (2006). Exposure to Altered Gravity During Specific Developmental Periods Differentially Affects Growth, Development, the Cerebellum and Motor Functions in Male and Female Rats. Adv. Space Res..

[13] Sajdel-Sulkowska EM, Nguon K, Sulkowski ZL, Rosen GD, Baxter MG (2005). Purkinje cell loss accompanies motor impairment in rats developing at altered gravity. Neuroreport.

[14] Sugiyama H, Ito I, Hirono C (1987). A new type of glutamate receptor linked to inositol phospholipid metabolism. Nat..

[15] Martin LJ, Blackstone CD, Huganir RL, Price DL (1992). Cellular localization of a metabotropic glutamate receptor in rat brain. Neuron.

[16] Hartmann J (2008). TRPC3 channels are required for synaptic transmission and motor coordination. Neuron.

[17] Hartmann J (2014). STIM1 controls neuronal Ca(2)(+) signaling, mGluR1-dependent synaptic transmission, and cerebellar motor behavior. Neuron.

[18] Kim SJ (2003). Activation of the TRPC1 cation channel by metabotropic glutamate receptor mGluR1. Nat..

[19] Aiba A (1994). Deficient cerebellar long-term depression and impaired motor learning in mGluR1 mutant mice. Cell.

[20] Finch EA, Augustine GJ (1998). Local calcium signalling by inositol-1, 4, 5-trisphosphate in Purkinje cell dendrites. Nat..

[21] Canepari M, Papageorgiou G, Corrie JE, Watkins C, Ogden D (2001). The conductance underlying the parallel fibre slow EPSP in rat cerebellar Purkinje neurones studied with photolytic release of L-glutamate. J. Physiol..

[22] Batchelor AM, Garthwaite J (1993). Novel synaptic potentials in cerebellar Purkinje cells: probable mediation by metabotropic glutamate receptors. Neuropharmacol..

[23] Ribeiro FM, Paquet M, Cregan SP, Ferguson SS (2010). Group I metabotropic glutamate receptor signalling and its implication in neurological disease. CNS Neurol. Disord. Drug. Targets.

[24] Crupi R, Impellizzeri D, Cuzzocrea S (2019). Role of Metabotropic Glutamate Receptors in Neurological Disorders. Front. Mol. Neurosci..

[25] Wang H (2016). Metabotropic Glutamate Receptors Induce a Form of LTP Controlled by Translation and Arc Signaling in the Hippocampus. J. neuroscience: Off. J. Soc. Neurosci..

[26] Yang S (2013). Integrity of mGluR-LTD in the associative/commissural inputs to CA3 correlates with successful aging in rats. J. neuroscience: Off. J. Soc. Neurosci..

[27] Yakusheva TA (2007). Purkinje cells in posterior cerebellar vermis encode motion in an inertial reference frame. Neuron.

[28] Tarnutzer AA, Wichmann W, Straumann D, Bockisch CJ (2015). The cerebellar nodulus: perceptual and ocular processing of graviceptive input. Ann. Neurol..

[29] Barmack NH (2003). Central vestibular system: vestibular nuclei and posterior cerebellum. Brain Res. Bull..

[30] Borisova T, Krisanova N, Himmelreich N (2004). Exposure of animals to artificial gravity conditions leads to the alteration of the glutamate release from rat cerebral hemispheres nerve terminals. Adv. Space Res..

[31] Dugue, G. P., Tihy, M., Gourevitch, B. & Lena, C. Cerebellar re-encoding of self-generated head movements. *Elife* **6**, 10.7554/eLife.26179 (2017). 10.7554/eLife.26179 PMC5489315 28608779

[32] Mackrous I, Carriot J, Jamali M, Cullen KE (2019). Cerebellar Prediction of the Dynamic Sensory Consequences of Gravity. Curr. Biol..

[33] Breniere Y, Bril B (1998). Development of postural control of gravity forces in children during the first 5 years of walking. Exp. Brain Res..

[34] Konczak J, Borutta M, Dichgans J (1997). The development of goal-directed reaching in infants. II. Learning to produce task-adequate patterns of joint torque. Exp. Brain Res..

[35] Ledebt A, Bril B, Breniere Y (1998). The build-up of anticipatory behaviour. An analysis of the development of gait initiation in children. Exp. Brain Res..

[36] Savelsbergh GJ, van der Kamp J (1994). The effect of body orientation to gravity on early infant reaching. J. Exp. Child. Psychol..

[37] Konczak J, Borutta M, Topka H, Dichgans J (1995). The development of goal-directed reaching in infants: hand trajectory formation and joint torque control. Exp. Brain Res..

[38] Mulavara AP (2012). Vestibular-somatosensory convergence in head movement control during locomotion after long-duration space flight. J. Vestib. Res..

